# Cutaneous Hyperpigmentation in Megaloblastic Anemia: a Five Year Retrospective Review

**DOI:** 10.4084/MJHID.2016.021

**Published:** 2016-04-10

**Authors:** Somanath Padhi, RajLaxmi Sarangi, Anita Ramdas, Kandasamy Ravichandran, Renu G’Boy Varghese, Thomas Alexander, George Kurian, Sudhagar Mookkappan

**Affiliations:** 1 Department of Pathology, Pondicherry Institute of Medical Sciences, Puducherry, India; 2 Department of Biochemistry, Pondicherry Institute of Medical Sciences, Puducherry, India; 3 Department of Biostatistics, Pondicherry Institute of Medical Sciences, Puducherry, India; 4 Department of Gastroenterology, Pondicherry Institute of Medical Sciences, Puducherry, India; 5 Department of General Medicine, Pondicherry Institute of Medical Sciences, Puducherry, India

## Abstract

**Background:**

Cutaneous hyperpigmentation is an often overlooked clinical sign in megaloblastic anemia (MA) which has been sporadically reported in the literature.

**Methods:**

We describe the bone marrow (BM) changes and clinicolaboratory characteristics of 25 of 198 adult cases (>16 years) with cutaneous hyperpigmentation who underwent BM evaluation for cytopenia (s).

**Results:**

Twenty-one of 25 cases (84%) had MA, while MA without hyperpigmentation occurred only in 12 of remainder 173 cases (P<0.001). Knuckle pad hyperpigmentation (KP) was noted in 16 (64%) cases; whereas 9 (36%) had diffuse brownish black discoloration (DP) of the palms and/or soles. Eighteen of 25 (72%) cases had pancytopenia (13 with KP) and 7 of 25 (28%) had bicytopenia (3 with KP). In addition, five cases (20%) presented with pyrexia. Of the 17 cases where data available, eleven were B_12_ deficient [<190 pg/ml; eight had severe deficiency (<100 pg/ml); ref.; 190–800pg/ml], while 4 had pure folate deficiency (< 4.0 ng/ml; ref.; 4–20ng/ml); and remainder 2 had combined B_12_ and folate deficiency. Compared to those with diffuse pigmentation; KP group had lower Hb (69.6 ± 24.2 vs. 86.3 ± 33.9 g/L), higher MCV (106.1 ±12.6 vs. 99.2 ± 7.6 fL), lower platelet count (50.9 ± 29.3 vs. 69.6 ± 36.5 × 10^9^/L), and lower median B_12_ [100.0 (30.0 – 822.0) vs. 316.0 (142.0 – 1617.3) pg/ml] (P>0.05). In six cases where follow-up data were available, there was a significant reversal of hyperpigmentation at 12 weeks following parenteral cobalamin therapy. In all five cases with pyrexia, fever subsided after 24 to 72 hours following administration of parenteral cobalamin therapy.

**Conclusion:**

Cutaneous hyperpigmentation and cytopenia (s) are strongly associated with megaloblastic anemia. Knuckle pad hyperpigmentation is much more frequent than diffuse pigmentation of the palms and/or soles in such patents. A nonsignificant trend towards a greater degree of MA was found in cases with pigmentation of the knuckles.

## Introduction

Megaloblastic anemia (MA) is a heterogeneous group of reversible bone marrow failure syndromes characterized by a variable degree of peripheral blood cytopenia (s) in the presence of a normo or hypercellular bone marrow. The hallmark pathophysiologic mechanism of MA is an impairment of DNA synthesis in all nucleated cells secondary to vitamin B_12_ (B_12_) and/or folate deficiency, resulting in nuclear-cytoplasmic asynchrony; distinctive megaloblastic changes, increased apoptosis, and ineffective hematopoiesis in the bone marrow.^1^ The manifestations of MA are diverse and may range from nonspecific signs and symptoms of anemia to gastrointestinal disturbances and potentially fatal neuropsychiatric and cardiovascular disorders.^2^

Megaloblastic anemia is not uncommon in the Indian subcontinent as well as other parts of Asia with females and vegetarians being more susceptible to B_12_ deficiency. Various studies in the past have shown that occult B_12_ deficiency may be rather prevalent among Indian urban and rural population.^3^–^5^

Cutaneous manifestations associated with B_12_ deficiency include characteristic mucocutaneous hyperpigmentation (most common), vitiligo, angular cheilitis, and hair-nail changes, which are often missed or overlooked in early, asymptomatic phases of the disease.^6^ In this manuscript, we describe the association of cutaneous hyperpigmentation (CP) with bone marrow changes in a series of 25 cases from a tertiary center in South India with a correlation of hematological and biochemical parameters, and also present a concise review of relevant literature. Furthermore, the association of pyrexia in MA is also briefly highlighted.

## Materials and Methods

In this retrospective study the bone marrow records of all adult cases (> 16 years), who underwent bone marrow aspiration (BMA) and trephine biopsy (Bx) over the last five years (October 2010 to December 2015) in the Department of Pathology of our Institute, were reviewed for the presence of CP. Informed written consent had been obtained from each case prior to the BM procedure; and the study was approved by the Institutional Ethics Committee.

As a part of the protocol for BM procedure, CP was prospectively documented in the bone marrow record by one of the authors (SP), prior to performing the procedure. Two types of pigmentation were documented: 1) dominant brownish-black pigmentation over dorsal aspect of hands and/or feet with accentuation over interphalangeal joints and periungual areas [knuckle pad (KP) group], and 2) diffuse and/or patchy, macular, dusky brownish-black discoloration/pigmentation over palms and/or soles (diffuse pigmentation (DP) group). Nature of BMA and gross appearance of Bx core were also recorded in each case. Data pertaining to routine hematological parameters such as hemoglobin (Hb), mean corpuscular volume (MCV), total leukocyte count (TLC), total platelet count (Plt), mean corpuscular hemoglobin (MCH), mean corpuscular hemoglobin concentration (MCHC), and peripheral blood smear (PBS) findings were collected from laboratory records in each case at the time of BM procedure.

BMA smears and Bx sections of all the above cases were retrieved from the departmental archives. Two pathologists (AR, RGV) who were blinded to the presence and nature of the pigmentation, reviewed the slides for the presence of definite megaloblasts. In each case, two MGG (May Grunewald Giemsa) and one Prussian blue (Perl stain) stained BMA smears were available for morphological study and assessment of the iron stores, respectively. A definitive diagnosis of MA was rendered with a constellation of PBS and BM findings such as erythroid hyperplasia, altered myeloid to erythroid ratio (M:E), presence of definite megaloblasts with sieve-like chromatin, nuclear-cytoplasmic asynchrony, giant abnormal shaped metamyelocytes and/or band forms, and/or abnormal (multinucleated) megakaryocytes with adequate (1–3+) or increased (≥4+) iron stores. A diagnosis of dimorphic marrow picture was rendered when there was admixture of both megaloblast and micronormoblast (with reduced hemoglobinization) and reduced (1+) or absent (0) iron store on Perl stain.^7^ When definite megaloblasts were not seen or very sparse, but dyspoietic changes were present, a possibility of macronormoblastic erythroid maturation with dyspoiesis was reported. Furthermore, those with dyspoietic changes and increased iron stores (≥4+) were also screened for the presence of ringed sideroblasts for a possible diagnosis of myelodysplastic syndrome (MDS) in the appropriate clinical setting.

Bone marrow changes were then correlated with the presence or absence and nature of hyperpigmentation, and serum biochemical parameters such as serum B_12_, folate, ferritin, and iron levels (wherever available). Serum B_12_ (ref.: 190 to 800 pg/ml), folate (ref.: 4 to 20 ng/ml), and ferritin levels [ref.: 30 to 400 ng/ml (males), 15 to 150 ng/ml (females)] were measured by electrochemiluminescence immunoassay (ECLIA) technique in Cobas e411 automated analyzer (Roche, Germany). Serum iron [ref.: 50 to 168 μg/dl (males), 35 to 145 μg/dl (females)] was measured in a semi-auto analyzer (Microlab 300, Merck) by using commercially available kits. Cases with B_12_ level <190 pg/ml were considered as B_12_deficient and those with levels< 100 pg/ml were considered as severe B_12_ deficiency [2]. Similarly, cases with folate levels < 4ng/ml were considered as folate deficient. Cases with serum ferritin < 30 ng/ml were considered as depleted iron stores; and those with < 15 ng/ml were considered as a severe iron deficiency.

Demographic profile, place of origin, dietary habits, history of prior illness and medications (if any), provisional clinical diagnoses, relevant clinical, microbiological and/or serological data were collected from the case records. All cases where a diagnosis of MA was made received parenteral (intramuscular) cyanocobalamin (1000 μg/day) for 7 days, followed by a weekly dose of the same for a minimum of 10 to 12 weeks. Those with dimorphic anemia were prescribed both parenteral and/or oral cobalamin in addition to hematinics and multivitamins.

### Statistical analysis

Continuous variables were described by mean (± SD) and Box plot were used to highlight the distribution of variables between two groups. Comparison between two groups was made using Fisher’s exact method for categorical variables, student t-test for normally distributed continuous variables, and Mann-Whitney test for not normally distributed continuous variables. All the tests were 2 sided; and a P value less than 0.05 was considered as statistically significant. SPSS software (version 20.0) was used for analyzing the data.

## Results

During the study period, a total of 304 patients underwent a BM examination, out of which 198 were personally performed by one of the authors (SP). All of these cases had been assessed for the presence or absence of CP at the time of the BM examination and hence were included in the analysis. CP was documented in 25 of these 198 patients (12.6%).

The demographic and clinico-laboratory characteristics of all 25 cases are presented in Tables 1 and 2. There were 16 males (64%) and 9 females (36%) with a mean age of 41.2 ± 16.7 years. Among the cases, 21 (84%) were dark skinned South Indians (in and around Puducherry). Seventeen of 25 (68%) were taking a mixed diet while only 8 (32%) were vegetarians. Seven of 16 (43.7%) males and none of the females had a history of alcohol abuse. Fatigue was the most common clinical presentation noted in 15/25 (60%) cases; 3/25 (12.0%) were diagnosed cases of autoimmune hepatitis on periodic follow-up with maintenance drugs such as azathioprine and prednisolone, 3/25 (12%) had evidence of gastric atrophy (anti-intrinsic factor antibody positive in one), 5 (20%) presented with fever, 2 (8%) had history of diarrhoea, 1 (4%) was a case of non-alcoholic steatohepatitis (NASH) secondary to uncontrolled type 2 diabetes mellitus, and 1 (4%) was a case of paranoid schizophrenia on olanzapine.

**Figure 1:**
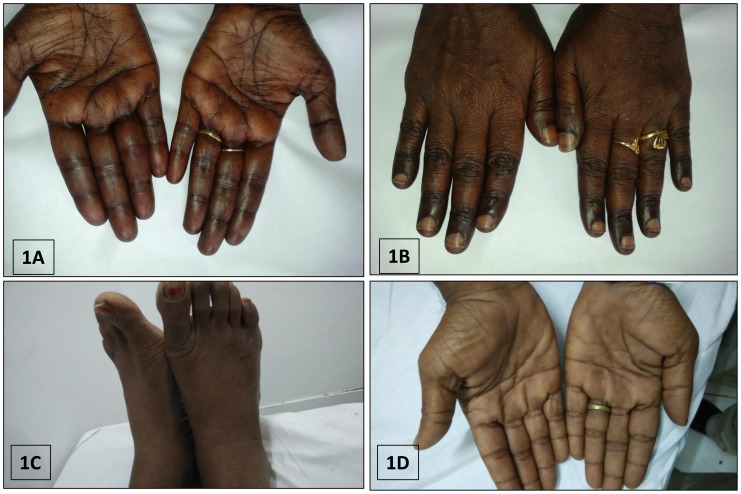
Peculiar cutaneous hyperpigmentation from cases with megaloblastic anemia: typical diffuse, brownish-black discoloration of the palms (**1A**), knuckle pad hyperpigmentation in the dorsum of hands (**1B**) in a case of megaloblastic anemia (prior to therapy) in a 52 year old female who had gastric atrophy, proven on endoscopic biopsy (case no;18, Table 2). This patient had pancytopenia, macrocytosis (MCV; 116fL), and severe B_12_ deficiency (55pg/ml). Note the reversal of pigmentation (**4A, 4B**) in the same patient, 12 weeks after initiation of parenteral cyanocobalamin therapy. Diffuse brownish-black pigmentation over dorsal aspect of feet (**1C**) and dusky, brownish-black discoloration of palms with accentuation of palmar creases (**1D**) in a 52 year old vegetarian male with fever, jaundice, pancytopenia, macrocytosis (MCV; 115fL), and florid megaloblastic anemia proven on bone marrow examination (B_12_ and folate assay not done) (case no; 23, Table 2).

The association of MA with CP was highly significant (P<0.001) with 21 (out of 25) patients having MA [20 had pure MA and 1 had mixed MA and iron deficiency anemia (IDA)] as compared to only 12 patients with MA (5 had pure MA and 7 had mixed MA and IDA) among 173 patients without pigmentation (Table 3). Prominent knuckle pad hyperpigmentation (KP) was documented in 16 of 25 (64%) cases whereas 9/25 (36%) cases had patchy or diffuse, dusky, brownish-black pigmentation over palms and/or soles with accentuation over palmar creases (DP) (Figures: 1A, 1B, 1C, 1D). Eighteen of 25 (72%) had pancytopenia (13 with KP, 5 with DP) and 7 (28%) had bicytopenia (3 with KP, 4 with DP). The mean Hb, MCV, TLC, and Plt were 75.4 ± 28.4 g/L, 103.7 ± 11.4 fL, 3.2 ± 1.2 × 10^9^/L, and 57.4 ± 32.5 × 10^9^/L, respectively.

**Figure 2:**
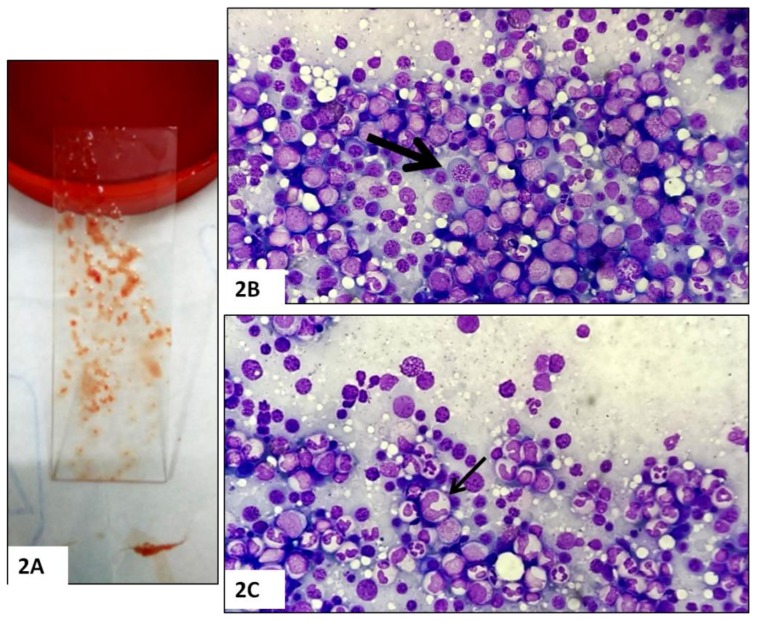
Bone marrow aspirate in megaloblastic anemia with cutaneous hyperpigmentation Note the richly particulate bone marrow aspirate (2A) obtained during bone marrow procedure in cases with cutaneous hyperpigmentation and cytopenia (s). Bone marrow aspirate smears demonstrating erythroid hyperplasia and megaloblasts with sieve-like nuclear chromatin (2B, thick arrow) and giant, abnormal shaped stab forms (2C, thin arrow). These findings were consistent with a diagnosis of megaloblastic anemia (May Grunewald Giemsa, ×400).

The marrow findings were consistent with MA in 20/25 (80%) cases [severe in 12, moderate in 5, and mild/focal in 3); dimorphic anemia with both megaloblasts and micronormoblasts in 1 case (4%); and dyspoietic changes with ringed sideroblast (3% of erythroid nuclei) suggestive of MDS was noted in one case (4%) (Figures: 2A, 2B, 2C). Nonspecific marrow findings and dyspoietic erythroid changes in the form of either macronormoblastic erythroid maturation, binucleation, nuclear budding, internuclear bridging were described in remainder 3 (12%) cases. There was no evidence of any granulomas, necrosis, organisms, hemoparasites, or any malignancy noted in any of the cases on the bone marrow. Detailed systemic, radiological, and endocrinological examinations were unremarkable in all cases and blood and BMA culture did not reveal any growth in cases with pyrexia.

Serum B_12_ and folate levels were available in 17/25 cases. It was found that 11/17 (64.7%) were B_12_ deficient (<190 pg/ml) among which 8 had severe B_12_ deficiency (<100 pg/ml); 4/17 (23.5%) were folate deficient (< 4 ng/ml); and 2/17 (11.8%) had combined B_12_ and folate deficiency.

**Figure 3:**
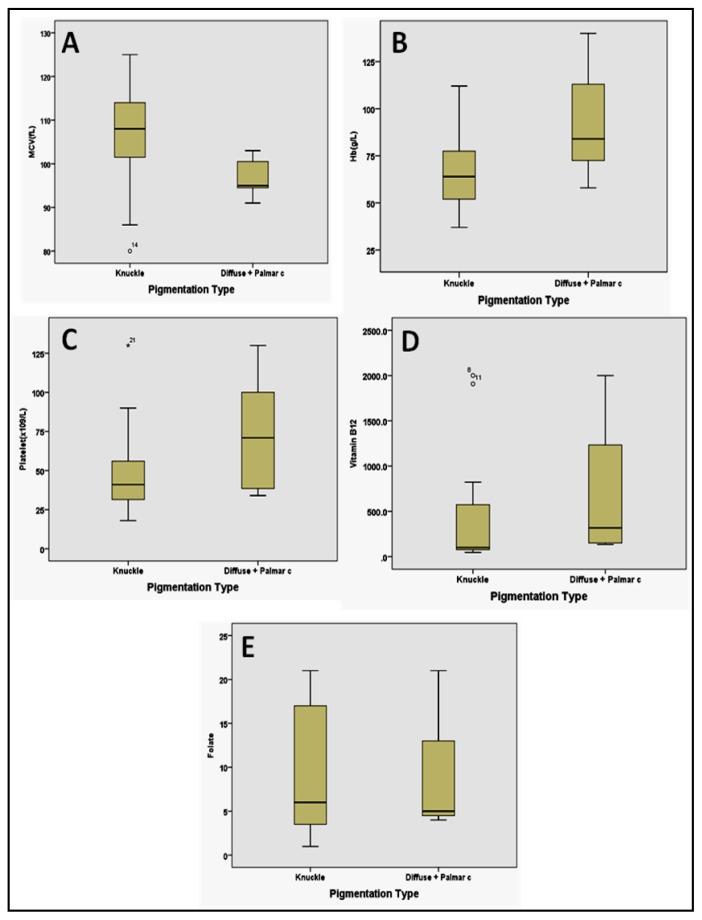
Box plot diagram depicting the comparison of median (50^th^ quartile, black horizontal line) and interquartile (25^th^ to 75^th^) range of mean corpuscular volume (MCV) (A), Hb (B), Platelets (C), B_12_ (D), and folate (E) levels among two groups of pigmentation [knuckle pad (KP) vs diffuse type (DP)]. Note that the median and interquartile range of Hb, Platelets, and serum B_12_ were lower in the KP group than in the DP group; whereas the median and interquartile range of MCV were higher in KP group than the DP group. Also note that the group with DP has a wider B_12_ value compared to the KP group (D). The median value of serum folate was similar among two groups.

KP group had lower Hb (69.6 ± 24.2 vs. 86.3 ± 33.9 g/L, respectively, P=0.19), higher MCV (106.1 ± 12.6 vs. 99.2 ± 7.6 fL, respectively, P=0.18), lower Plt (50.9 ± 29.3 vs. 69.6 ± 36.5 × 10^9^/L, respectively, P=0.15) than the DP group (Table 4). Similarly, the median (50^th^ quartile) and interquartile (25^th^ to 75^th^ quartile) range for Hb and Plt were lower in the former group than the latter [64.0 (52.0 – 80.0) vs 81.0 (60.3 – 116.0) g/L; 41.0 (30.0 – 56.0) vs. 58.0 (36.8 – 100.0) × 10^9^/L, respectively]. However, median and interquartile range for MCV were higher between two groups [108.0 (99.0 – 114.0) vs. 97.0 (94.3–102.8) fL, respectively, P>0.05]. Median and interquartile range of serum B_12_ values in the KP group was lesser [100.0 (30 – 822.0) pg/ml] compared to the later group [316.0 (142.0 – 1617.3) pg/ml] (P = 0.17); whereas median serum folate levels were similar between two groups [6.0 (3.0 – 18.0) vs. 5.0 (4.3 – 17.0) ng/ml, respectively, P=0.79] (Table 4, Figures 3A–3E).

**Figure 4:**
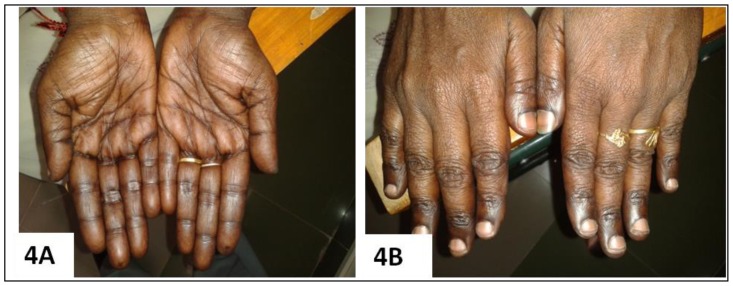
Reversal of hyperpigmentation in the patient of figure 1A and 1B, 12 weeks after initiation of parenteral cyanocobalamin therapy.

Six of 25 cases where follow-up data were available, showed dramatic improvement (> 85%) in their hyperpigmentation following 12 weeks B_12_ therapy (Figures 4A and 4B), whereas the rest were lost to follow-up. In all the five cases who presented with pyrexia, their fever subsided following day 3 to 4 of starting parenteral cyanocobalamin therapy.

## Discussion

Megaloblastic anemia is a multisystemic disorder where hematological and neuropsychiatric manifestations usually dominate the clinical picture. In 1944, Dr Bramwell Cook first observed that hyperpigmentation of the skin was associated with a macrocytic anemia and that both it and the anemia responded to crude liver extract.^8^ Since then, there have been sporadic case reports and small case series with descriptions of the peculiar skin-hair-nail changes in patients with megaloblastic anemia.^9^–^13^ In our series, we observed a strong association of cutaneous hyperpigmentation with megaloblastic anemia, and knuckle pad pigmentation was much more frequent than the diffuse type.

We also observed a good correlation between the presence of CP and BM changes with serum biochemical parameters. Barring one case of dimorphic anemia, none of the cases with hyperpigmentation had depleted marrow iron stores which in turn correlated well with the normal or increased serum ferritin levels in 24/25 cases. A greater proportion of our cases were B_12_ deficient (<190 pg/ml); and eight had significant B_12_ deficiency (<100 pg/ml). Cases with KP were associated with a greater degree of B_12_ deficiency, macrocytosis, and pancytopenia; though this lacked statistical significance. Our observation was in accordance with that published in the literature.^8^,^9^,^11^ Though majority (68%) of our subjects were on a mixed diet, a higher proportion of MA diagnosis suggests that factors other than poor intake like impaired absorption might be responsible for B_12_ and/or folate deficiency; and alcohol abuse was a contributing factor among our male subjects. However, the presence of hyperpigmentation in a case of suspected MDS as well as in other 3 cases with reactive marrow changes contradicts the above hypothesis. Furthermore, an interesting aspect of our cohort was the fact that 5 of 21 cases (23.8%) with MA presented with pyrexia requiring extensive work up. However, in all cases, fever subsided within 24 to 72 hours of initiation of parenteral cyanocobalamin therapy. This reinforces our previous observation that florid ineffective hematopoiesis in MA, in conjunction with other but yet unidentified mechanisms, might be the underlying cause for such phenomenon.^14^,^15^

A brief comparative review of literature regarding mucocutaneous hyperpigmentation in MA and/or B_12_ deficiency is presented in Table 5.^8^–^13^,^15^ In 1963, Baker and colleagues described characteristic reversible brownish-black pigmentation over dorsal aspect of interphalangeal joints of hands and feet (KP) in a large series of 21 South Indian subjects with MA (15 adults, 6 infants/children). Malabsorption was the commonest cause of B_12_ deficiency in that series; and the mean serum B_12_ level among cases was very low (49 pg/ml) by using microbiological assay method.^8^ Similarly, reversible Addisonian type of mucocutaneous hyperpigmentation and nail changes were also reported in a dermatologic setting among nine South Indian patients with biochemical evidence of B_12_ deficiency.^9^ Aaron et al^10^ described mucocutaneous changes as a significant physical finding in 26 of 63 patients (41%) with neurological manifestations secondary to B_12_ deficiency. Cutaneous hyperpigmentation was found to be the most common (19% of cases) whereas hair changes and vitiligo were described in 9% and 3% of cases, respectively. Hyperpigmentation did not show any correlation with duration of symptoms, severity of megaloblastosis, and MCV; and follow-up data of skin changes were not reported in that series.^10^ A recent prospective study from Turkey^11^ recruited 57 pediatric subjects (mean age; 12.75 ± 4.75 months) of which 49 (86%) were exclusively breastfed. A higher proportion (63%) of cases had a severe B_12_ deficiency (<100 pg/ml); and 44 of 57 mothers were also B_12_ deficient (<200 pg/ml). Forty-nine of 57 (86%) babies had CP and 40 (70%) had atrophic glossitis. On serial follow-up at the end of 1 week, 4weeks, and 12 weeks, there was a dramatic improvement in mucocutaneous changes at 12 weeks following parenteral cobalamin therapy.^11^ Similarly most of the other published reports have also noted a dramatic improvement or near complete reversal of the pigmentary changes following 8 to 12 weeks of parenteral cobalamin therapy.^12^,^13^,^15^

**Figure 5:**
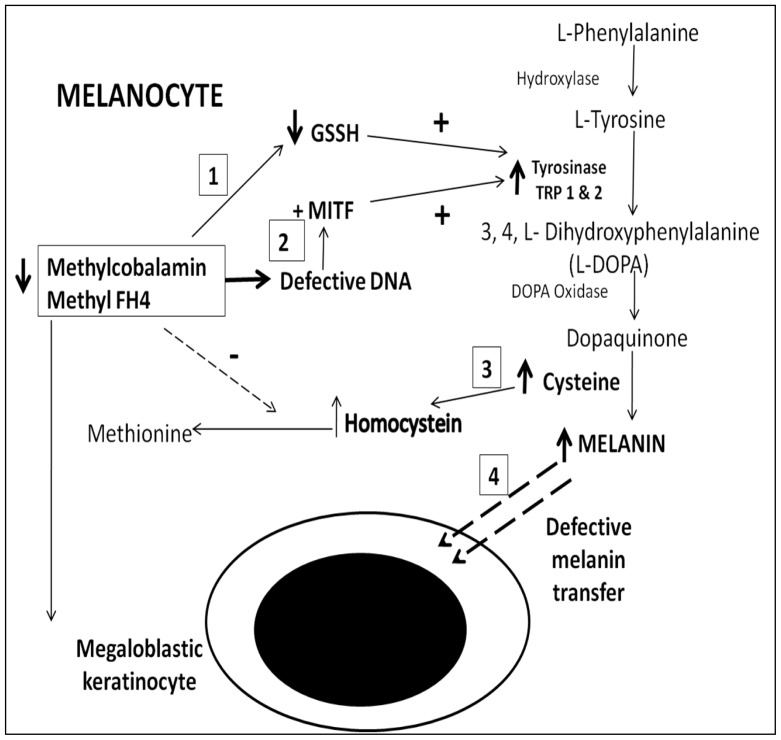
The postulated biochemical mechanism of hyperpigmentation in megaloblastic anemia.^6^,^16^ The 4 most accepted mechanisms involved are: 1) low methylcobalamin level in melanocytes leads to reduced level of reduced glutathione (GSSH); which in turn activates Tyrosinase enzyme in melanin synthesis pathway, 2) defective DNA synthesis activates Microphthalmia-associated transcription factor (MITF); which causes activation of both Tyrosinase and Tyrosinase related protein 1 and 2 (TRP 1and 2),^16^ 3) hyperhomocysteinemia leads to accumulation of cysteine leading to increased melanin synthesis, 4) defective melanin transfer from the melanocytes to adjacent megaloblastic keratinocytes. Increased angiogenesis secondary to upregulation of dermal vascular endothelial growth factor (VEGF) may also lead to increased pigmentation.^18^ Both histopathologic and ultrastructural studies have postulated that hyperpigmentation is due to increased number of basal melanocytes as well as increased melanosomes.^19^

The pathophysiologic mechanism associated with hyperpigmentation in B_12_ and/or folate deficiency seems to be complex and is poorly understood.^6^,^16^ However, the most accepted hypotheses are i) increased melanin synthesis, and ii) defective melanin transfer from melanocytes to adjacent megaloblastic keratinocytes (Figure 5).

Reduced methylcobalamin causes a reduction in intracellular reduced glutathione (GSSH) which in turn, activates Tyrosinase enzyme in the L-phenylalanine - L-tyrosine - melanin pathway. Also, defective DNA synthesis leads to activation of micro-ophthalmia associated transcription factor (MITF), which upregulates both Tyrosinase and Tyrosinase related proteins (TRP 1 and 2).^16^ Furthermore, hyperhomocysteinemia in B_12_deficiency leads to increased cysteine level augmenting melanin synthesis. Both histopathologic and ultrastructural studies in skin biopsies have suggested that hyperpigmentation is not due to a defect in melanin transport but is secondary to an increase in melanin synthesis.^17^,^18^ Moreover, increased angiogenesis secondary to upregulation of vascular endothelial growth factor (VEGF) has also been postulated to be responsible for the reddish brown discoloration seen in some cases.^19^ However, the reason for the localized regional hyperpigmentation over the knuckle regions and greater prevalence among dark skinned individuals remains an enigma. It is open to speculation whether genetic and racial differences are responsible for this peculiar phenomenon.

The present study has certain limitations. 1) The retrospective nature of the survey led to the fact that CP was not assessed in all patients undergoing a bone marrow evaluation, resulting in a limited sample size. Not all patients with MA diagnosed on the basis of hematological parameters patients would have had a bone marrow examination performed. We also do not have data as to how many patients who presented to the hospital during this period had the typical cutaneous hyperpigmentation. 2) A lack of correlation of MA with biochemical parameters was found in up to 40% of cases. This can be explained by the poor sensitivity and wider reference range of the assay technique (ECLIA) used in our laboratory, which generally gives higher values as compared to the more accurate microbial assay technique.^2^ 3) Lastly, lack of follow-up in the 15 of 21 cases with MA was a major limitation in our study.

## Conclusions

This is the first study that has systematically evaluated cutaneous hyperpigmentation among patients undergoing a bone marrow examination in which a definite association with megaloblastic anemia was observed. The present study reinforces the fact that cutaneous hyperpigmentation and pyrexia are helpful clinical signs in megaloblastic anemia. These in the presence of cytopenia (s) are reliable markers in the megaloblastic anemia and clinicians should be aware of these valuable clinical signs.

## Figures and Tables

**Table 1:** Base line characteristics of cases with cutaneous hyperpigmentation which was documented during bone marrow procedure (October 2010–December 2015).

Characteristic	Value
**Number of cases**	25
• Male (n, %)	16 (64)
• Female (n, %)	9 (36)
**Mean age in years**	41.2 ± 16.7
**Dietary habit (n, %)**	
• Vegetarian diet	8 (32)
• Mixed diet	17 (68)
• H/o alcohol intake (males only)	7/16 (43.7)
• On medication	4 (16)
**Presentation (n, %)**	
• Fatigue/myalgia	17 (68)
• Autoimmune hepatitis on Azn and Pdn	3 (12)
• Atrophic gastritis (biopsy proved)	3 (12)
• Fever	5 (20)
• Diarrhoea	2 (8)
• Type 2 diabetes mellitus with NASH	1 (4)
• Schizophrenia on Olanzapine	1 (4)
**Pigmentation (n, %)**	
• Knuckle padח	16 (64)
• Diffuse palm/sole ± accentuation of crease	9 (36)
**Laboratory parameter**	
• Bicytopenia (n, %)	7 (28)
• Pancytopenia (n, %)	18 (72)
• Mean corpuscular volume (mean ± SD)	103.7 ± 11.4 fL
• Hemoglobin (mean ± SD)	75.4 ± 28.4 g/L
• Total leukocyte count (mean ± SD)	3.2 ± 1.2 × 10^9^/L
• Mean platelet count (mean ± SD)	57.4 ± 32.5 × 10^9^/L
**Bone marrow changes (n, %)**	
• Erythroid hyperplasia	25 (100)
• Pure megaloblastic anemia	20 (80)
• Dimorphic†	1 (4)
• Macronormoblastic	3 (13.0)
• With ringed sideroblast (3%)	1 (4)
• Iron stores (Perl stain) (≥2+)	24 (96)
**Serum vitamin B_12_** **(ECLIA)**‡ **(n, %)**	
• <190 pg/ml	11 (44)
• ≤ 100 pg/ml	8/11 (72.7)
• 190–800 pg/ml	2 (8)
• > 800 pg/ml	4 (16)
• Not done	8 (32)
**Serum folate (ECLIA)**‡‡ **(n, %)**	
• < 4 ng/ml	6 (24)
• 4–20 ng/ml	8 (32)
• >20 ng/ml	3 (12)
• Not done	8 (32)

**Abbreviation:** Azn; azathioprine, Pdn; prednisolone, NASH; non-alcoholic steatohepatitis

ח ; dorsal aspect of interphalangeal joints of hands and/or feet with periungual accentuation,

† ; both megaloblastic and micronormoblastic erythroid maturation, ECLIA; electrochemiluminescence immunoassay;

‡ ; reference range (190–800 pg/ml),

‡‡ ; reference range (4–20 ng/ml). Note: B_12_ level < 190 was considered B_12_ deficient, levels < 100 pg/ml was considered severe B_12_ deficiency. Similarly, folate level < 4ng/ml was considered as folate deficient. Note: of all 6 cases with low folate, 2 had associated B_12_ deficiency, and pure folate deficiency was noted in four cases.

**Table 2:** Cutaneous hyperpigmentation: clinicolaboratory profile of 25 cases (October 2010–December 2015).

Sl. No	Age (yrs), gender	Presentation	Dietary habit	Pigmentation in hand	Cytopenia (s)	Hb (g/L)	MCV (fL)	TLC (×10^9^/L)	Platelet (×10^9^/L)	M:E ratio	Erythroid maturation	B_12_ (pg/ml) (190–800) ¶	Folate (ng/ml) (4–20)¶	BM iron stores (Perl stain)
1	27, F	Fatigue, autoimmune hepatitis, on Azn+Pdn	Mixed	Diffuse (palm)	Pancytopenia	107	99	2.5	35	1:1	Megaloblastic	166	4.8	3+
2	18, F	Fatigue	Veg.	Knuckle pad	Pancytopenia	52	105	3.1	56	1:2	Megaloblastic	97	1.4	3+
3	33, F	Fatigue, fever	Mixed	Diffuse (palm)	Pancytopenia	78	102	4.7	42	1:2	Megaloblastic	466	>20	2+
4	27, M	Fatigue	Mixed, alcoholic	Knuckle pad	Bicytopenia	80	114	5.7	30	1:2.5	Megaloblastic	Not done	Not done	2+
5	24, M	Fever, diarrhoea	Mixed	Diffuse (palm)	Bicytopenia	140	103	2.6	100	1:2	Megaloblastic	134	5.2	2+
6	69, M	Nonspecific	Mixed, alcohol	Diffuse (palm)	Pancytopenia	11.9	95	2.9	71	1.4:1	Macro normoblastic	>2000	4.2	4+
7	14, F	Autoimmune hepatitis, on Azn+Pdn	Mixed	Knuckle pad + diffuse (palm)	Pancytopenia	112	99	3	41	1:1.6	Megaloblastic	Not done	Not done	2+
8	43, M	Fatigue	Mixed, Alcoholic	Knuckle pad	Pancytopenia	64	106	2.8	56	1:1	Megaloblastic	>2000	>20	2+
9	65, M	Nonspecific	Mixed	Knuckle pad	Pancytopenia	109	113	3	33	1:1.7	Megaloblastic	Not done	Not done	3+
10	19, M	Fatigue	Mixed	Knuckle pad	Pancytopenia	41	91	2.6	36	1:4	Megaloblastic	326	3.2	3+
11	36, M	Fatigue, autoimmune hepatitis on Azn+Pdn	Mixed	Knuckle pad	Pancytopenia	52	86	0.9	18	1:6:1	Macro normoblastic	1908	15.5	2+
12	63, F	Severe fatigue	Veg	Palmar crease	Pancytopenia	84	95	3.6	34	1:3	Megaloblastic	Not done	Not done	3+
13	36, M	Nonspecific	Mixed, alcoholic	Diffuse + palmar crease	Bicytopenia	58	91	4	130	1:1	Macro normoblastic	Not done	Not done	2+
14	37, F	Fatigue	Mixed	Knuckle pad	Bicytopenia	108	80	5.2	30	1:1	Dimormic	93	12.3	1+
15	45, M	Fatigue, atrophic gastritis, diarrhoea	Mixed	Knuckle pad	Pancytopenia	75	110	3.5	80	1:3	Megaloblastic	100	4.3	4+
16	51, F	Fatigue	Veg	Knuckle pad	Pancytopenia	74	114	2.6	50	1:2	Megaloblastic	59.6	3.9	4+
17	70, M	Nonspecific	Veg	Knuckle pad	Pancytopenia	72	125	4.5	50	1:3	? MDS	822	>20	2+ (Ring sideroblast 3%)
18	52, F	Fatigue, atrophic gastritis	Veg	Knuckle pad	Pancytopenia	64	116	2.5	90	1:1.5	Megaloblastic	55	18.3	3+
19	33, M	Schizophrenia, Olanzapine	Mixed	Knuckle pad	Pancytopenia	55	108	1.1	33	1:1.3	Megaloblastic	103	1.8	3+
20	28, M	Fever, fatigue, jaundice	Mixed, alcoholic	Knuckle pad	Pancytopenia	49	104	1.8	30	1:5	Megaloblastic	45	6	2+
21	55, M	Generalised myalgia	Mixed, alcoholic	Knuckle pad	Bicytopenia	37	120	4.9	130	1:4	Megaloblastic	Not done	Not done	2+
22	50, M	Type 2 diabetes, NASH, fever	Mixed, alcoholic	Diffuse (palm)	Bicytopenia	67	94	2.7	100	1:1.2	Megaloblastic	Not done	Not done	2+
23	52, M	Fever, jaundice	Veg	Diffuse (feet)	Pancytopenia	37	115	2.8	45	1:5	Megaloblastic	Not done	Not done	3+
24	46, M	Fever, abd. pain, gastric atrophy+ Fatigue	Mixed	Generalised	Pancytopenia	57	124	4.1	100	1:3	Megaloblastic	46.7	14.4	4+
25	16, F		Veg	Diffuse (palms and soles)	Bicytopenia	37	112	5.8	140	1:2.5	Megaloblastic	<30	8.8	3+

**Abbreviations:** F; female, M; male, Azn; azathioprine, Pdn; prednisolone, NASH; non-alcoholic steatohepatitis, Veg; vegetarian, Hb; hemoglobin, MCV; mean corpuscular volume, TLC; total leukocyte count, M: E; myeloid to erythroid ratio, MDS; myelodysplastic syndrome, BM; one marrow aspirate,

¶ ; assay by electrochemiluminescence method. Note: six cases (case nos. 16, 18, 19, 20, 23, 24) showed a dramatic (> 85%) improvement in their hyperpigmentation following 12 week B_12_ therapy whereas follow-up data were not available in 18 cases; and case no 25 is a newly diagnosed case and presently under follow-up.

**Table 3:** Association of megaloblastic anemia and presence of cutaneous hyperpigmentation.

		Total bone marrows performed = 198		
	
		Pigmentation present (N=25)	Pigmentation NOT present (N=173)	
Megaloblastic anemia	Yes	20 (80%)	5 (3%)	P<0.001#
	No	4 (16%)	161 (93%)	
Combined megaloblastic and iron deficiency (dimorphic) anemia		1 (4 %)	7 (4%)	

# ; Fischer’s exact test

**Table 4:** Comparative characteristics between two groups of cases with cutaneous hyperpigmentation.

Characteristic	KP (n=16)	DP (n=9)	p value
Gender (male/female, n/n)	11/5	5/4	1.00
Mixed diet	12	5	1.00
Alcohol intake*	4	3	0.61
Pancytopenia	13	5	0.62
Mean corpuscular volume (fL)			
• Mean (± SD)	106.1 (12.6)	99.2 (7.6)	0.18
• Median†‡	108.0 (99.0–114.0)	97.0 (94.3–102.8)	
Hemoglobin (g/L)			
• Mean (± SD)	69.6 (24.2)	86.3 (33.9)	0.19
• Median†‡	64.0 (52.0–80.0)	81.0 (60.3–116.0)	
Platelet count (×10^9^/L)			
• Mean (± SD)	50.9 (29.3)	69.6 (36.5)	0.15
• Median†‡	41.0 (30.0–56.0)	58.0 (36.8–100.0)	
Serum vitamin B_12_ (pg/ml) (median)†‡	100.0 (30.0 – 822.0)	316.0 (142.0 – 1617.3)	0.17
Serum folate (ng/ml) (median)†‡	6.0 (3.0 – 18.0)	5.0 (4.3 – 17.0)	0.79

* ; all were males,

† interquartile range (25^th^ to 75^th^ quartile range),

‡ ; refer to Box Plot diagram (Figures 3A–3E),

KP; knuckle pad hyperpigmentation in hands and/or feet, DP; diffuse pigmentation in palms and/or soles.

**Table 5:** Review of literature related to hyperpigmentation in vitamin B_12_ and/or folate deficiency.

Author, year, place, ref.	Number (male/female), age	Nature of pigmentation	Etiology of B_12_ deficiency	Laboratory parameters	Management, follow-up, remarks
Baker et al,^8^ 1963, Vellore, India	21 (15/6), 15 adults, 6 infants/children	Knuckle pad (KP), brownish black	Malabsorption syndrome	19/21; megaloblastic anemia in BM studies; 21/21; low B_12_ (mean; 49 pg/ml) (microbiological assay); Hb; 29–143g/L	Inj. cobalamine Improvement: Infants; 3 weeks, adults; 6–12 weeks.
Jithendriya et al,^9^ 2013, Bangalore, India	9 (7/2), 21–51 yrs	Addisonian type, face, oral mucosa, KP, palm, nail bed.	Vegetarian; 7/9	9/9; low B_12_ (50–93 pg/ml) 6/9; MCV ≥98 fL 9/9; normal cortisol	Oral cobalamin (1000μg/day); 9/9 80% reduction in pigmentation; 4 weeks.
Aaron et al,^10^ 2005, Vellore, India	63 (52/11), 46.2 yrs	26; skin and mucosal changes + 12 (19 %); hyperpigmentation + 3%; vitiligo +	Autoimmune gastritis; 19/25 Alcoholism; 10/63 Diarrhea; 6/63	35/39; low B_12_ 13/14; elevated homocysteine	No correlation of hyperpigmentation with duration of symptoms, severity of megaloblastosis, and mean corpuscular volume. Hyperpigmentation, no follow-up with therapy.
Demir et al,^11^ 2014, Turkey	57, 12.75 ± 4.75 months (6–24 months)	49/57 (86 %); hyperpigmentation+40/57 (70.17%); atrophic glossitis+ Extremities, KP, inner thigh, axilla, neck fold, genitals	Breast feeding only; 49/57 44/57 mothers; B_12_ < 200pg/ml	36/57 (63.15%); severe deficiency (<100 pg/ml)	Inj. cobalamin Dramatic improvement (87%) -12 weeks
Kannan et al,^12^ 2008, Tamil Nadu, India	1; 34 yrs, female, 2; 54 yrs, female	KP (feet)-Case 1 Diffuse (neck and back)- Case 2	Hyperthyroidism; case 1 Autoimmune gastritis; case-2	Case-1; low B_12_, normal MCV and Hb; Case-2; low B_12_, MCV-103 fL	Inj. cobalamin Improvement in 2 weeks- case 1 Improvement in 4 weeks- case 2
Santra et al,^13^ 2014, WB, India	45 yrs, female	Generalized hyperpigmentation	Vegetarian	Hb/MCV/B_12_: 39 g/L; 118fL; 81.3 pg/ml	Inj. hydroxycobalamin Improvement at 12 weeks.
Chakrabarty S,^15^ 2015, WB, India	62 yrs, male	KP, fever	Vegetarian H/o: gastrectomy for gastric carcinoma	Hb/B_12_/folate: 66g/L/76pg/ml/3.2 ng/ml	Inj. cobalamin Improvement; at 12 weeks.
Present series, 2015, Puducherry, India	25 (16/9) 41.2 ± 16.7	16/25; KP hyperpigmentation 9/25; diffuse hyperpigmentation 5/25; fever	8/25; vegetarian 17/25; mixed diet 7/25; alcohol abuse 4/25; drugs 3/25; atrophic gastritis	21/25; megaloblastic anemia in BM studies, 17/23; pancytopenia, 11/17; low B_12_ (<190 pg/ml), 8/11; very low B_12_ (<100 pg/ml)	Inj. cobalamin Dramatic improvement in 6 cases at 12 weeks. Fever subsided in all 5 cases
